# MLKL Deficiency Stabilizes RIP3 and Aggravates Myocardial Injury by Promoting Apoptosis and Pyroptosis

**DOI:** 10.3390/cimb48040380

**Published:** 2026-04-07

**Authors:** Ziguan Zhang, Zuheng Liu, Yilei Liu, Changqing Sun, Weihua Li, Wuyang Zheng

**Affiliations:** Xiamen Key Laboratory of Cardiac Electrophysiology, Department of Cardiology, Xiamen Institute of Cardiovascular Diseases, The First Affiliated Hospital of Xiamen University, School of Medicine, Xiamen University, Xiamen 361003, China

**Keywords:** MLKL, RIP3, myocardial infarction, apoptosis, pyroptosis

## Abstract

Regulated cardiomyocyte death is a central contributor to myocardial infarction (MI)-associated injury. Mixed lineage kinase domain-like protein (MLKL), a key effector of necroptosis, has been implicated in cardiovascular disease; however, its role in MI remains incompletely defined. MLKL expression was evaluated in hypoxia-treated cardiomyocytes, infarcted murine hearts, and human cardiac tissue. MLKL function was investigated using siRNA-mediated knockdown in neonatal mouse cardiomyocytes and genetic deletion in mice subjected to left anterior descending (LAD) coronary artery ligation. Apoptosis- and pyroptosis-related signaling were assessed by immunoblotting and immunostaining. RIP3 expression and regulation were examined at both protein and mRNA levels, and the RIP3 inhibitor GSK’872 was used to assess pathway dependence. MLKL expression was increased in hypoxic cardiomyocytes, infarcted mouse hearts, and human failing cardiac tissue. Unexpectedly, MLKL deficiency was associated with aggravated myocardial injury, impaired cardiac function, and increased fibrosis following MI. Mechanistically, MLKL deficiency was associated with increased RIP3 protein abundance without a corresponding increase in RIP3 mRNA, consistent with post-transcriptional regulation. Further analyses indicated that MLKL deficiency reduced RIP3 ubiquitination and impaired proteasome-mediated degradation, resulting in RIP3 stabilization. Elevated RIP3 levels were accompanied by increased expression of apoptosis- and pyroptosis-related proteins, particularly at early time points after MI. Pharmacological inhibition of RIP3 with GSK’872 was associated with reduced apoptosis- and pyroptosis-related signaling and improved cardiac function. MLKL deficiency is associated with stabilization of RIP3 and enhanced activation of apoptosis- and pyroptosis-related signaling following MI, contributing to aggravated myocardial injury. These findings support a regulatory role for the MLKL–RIP3 axis in cardiomyocyte death and suggest that targeting RIP3 may represent a potential therapeutic strategy in myocardial infarction.

## 1. Introduction

Myocardial infarction (MI) remains a major cause of morbidity and mortality worldwide and is a leading antecedent of adverse ventricular remodeling and subsequent heart failure (HF). Increasing evidence indicates that regulated cardiomyocyte death contributes substantially to post-MI injury and remodeling. Among these pathways, necroptosis has emerged as an important mediator of ischemic myocardial damage and a potential therapeutic target in cardiovascular disease [1].

Mixed lineage kinase domain-like protein (MLKL) is the canonical downstream effector of necroptosis and has therefore been widely viewed as a mediator of cardiomyocyte loss. However, the role of MLKL in the setting of MI may be more complex than simple execution of necroptotic death. Although previous preclinical studies have suggested that targeting the necroptotic pathway may confer cardioprotection [2], the specific contribution of MLKL to post-MI remodeling, and whether it exerts additional regulatory effects beyond terminal necroptosis execution, remain insufficiently defined.

In this study, we sought to systematically investigate the role of MLKL in myocardial infarction using complementary in vitro and in vivo approaches. Specifically, MLKL expression was evaluated in hypoxia-treated cardiomyocytes, infarcted murine hearts, and human cardiac tissue. The functional consequences of MLKL deficiency were examined using siRNA-mediated knockdown in neonatal mouse cardiomyocytes and genetic deletion in mice subjected to left anterior descending (LAD) coronary artery ligation. In addition, we explored the potential relationship between MLKL and RIP3, as well as downstream apoptosis- and pyroptosis-related signaling pathways, to better understand the contribution of MLKL to the regulation of cardiomyocyte death following myocardial infarction.

## 2. Method

Only male mice were used in this study to reduce potential variability associated with sex-dependent differences in post-MI remodeling.

### 2.1. Mice

In this study, MLKL^−/−^ mice on a C57BL/6 background donated by the Jiahuai Han Laboratory were used as experimental animals. All mice were housed in a specific pathogen-free (SPF) facility at the Xiamen University Experimental Animal Center under a 12 h light/dark cycle. Room temperature was maintained at 22–24 °C and humidity at 50–70%. Mice were monitored throughout the study, and humane endpoints were applied according to institutional animal care requirements. Animals exhibiting severe hypothermia or marked distress were euthanized by CO_2_ inhalation.

### 2.2. Left Anterior Descending (LAD) Coronary Artery Ligation Model of MI

Under general anesthesia, a left thoracotomy was performed through the fourth intercostal space to expose the heart and identify the LAD coronary artery. The LAD coronary artery was permanently ligated with an 8-0 polypropylene suture to induce myocardial infarction, as evidenced by immediate blanching of the anterior ventricular wall and ST-segment elevation on surface electrocardiography. The thoracic cavity was then closed in anatomical layers, and mice received postoperative analgesia along with prophylactic antibiotics to ensure effective pain control and infection prevention.

Additional procedural details: The LAD was ligated approximately 1–2 mm below the tip of the left atrial appendage. Animals were randomly assigned to experimental groups. Investigators performing infarct size measurement, fibrosis quantification, and echocardiographic analysis were blinded to genotype and treatment allocation during analysis. Animals that died during or immediately after surgery were excluded from subsequent analyses. Mice without successful induction of myocardial infarction were also excluded. All remaining animals were included in the final experimental analyses.

### 2.3. Histopathology

Histopathological analysis was performed to evaluate structural alterations in myocardial tissue following MI. After euthanization, heart tissues were carefully harvested and fixed in 10% neutral-buffered formalin for 24 h at room temperature. The fixed tissues were then embedded in paraffin, and serial sections of 4 µm sections thickness were cut and mounted on glass slides for further examination. The tissue sections underwent hematoxylin and eosin (H&E) staining according to standard protocols, using hematoxylin (CTS-1096, MXB Biotechnologies, Fuzhou, China) and eosin (ZLI-9613, ZSGB-BIO, Beijing, China) to evaluate general tissue architecture and inflammatory changes.

For immunohistochemical (IHC) analysis, paraffin-embedded myocardial sections were subjected to antigen retrieval by incubation in a 10 mmol/L sodium citrate buffer (pH 6.0) (W302600, Sigma, Hong Kong, China), supplemented with 0.05% Tween-20 (P1379-6X, Sigma) for 20 min at 95 °C. The sections were subsequently blocked using 2.5% normal horse serum (S-2012-50, Vector Laboratories, Newark, CA, USA) to minimize non-specific binding.

For detection of phosphorylated MLKL (p-MLKL, S345), selected sections were incubated overnight at 4 °C with primary anti-p-MLKL (S345) antibody (ab196436, Abcam, Cambridge, UK, dilution 1:1500). The sections were then incubated with horse anti-mouse/rabbit IgG (MP-7500, Vector Laboratories) for 1 h at room temperature.

In parallel, other sections were incubated overnight at 4 °C with an anti-cleaved Caspase-8 antibody (9429S, CST, Hong Kong, China, dilution 1:200) to assess apoptosis markers. Detection was achieved using biotinylated anti-mouse/rabbit IgG (BA-1400-2.1, Vector Laboratories) for 1 h at room temperature.

All immunohistochemical stained slides were evaluated under a Leica DM2500 (Wetzlar, Germany) microscope at Xiamen University. Representative images were captured using identical imaging settings across groups to ensure consistency in data acquisition.

### 2.4. Masson’s Trichrome Staining

Masson’s trichrome staining was utilized to assess myocardial fibrosis in tissue sections. Sample preparation, sectioning, and dehydration were carried out as described above for immunohistochemistry. Sections were rehydrated in distilled water and stained with hematoxylin for 5 min to visualize nuclei, followed by rinsing in running tap water. Slides were incubated with Biebrich scarlet-acid fuchsin solution for 10 min to stain cytoplasm and muscle fibers. After differentiation in phosphomolybdic-phosphotungstic acid (Weigert’s iron hematoxylin) for 2 min, the sections were counterstained with aniline blue for 10 min to highlight collagen fibers. Finally, the slides were dehydrated, cleared in xylene, and mounted with a resinous mounting medium.

Fibrosis quantification was performed using ImageJ software 1.53d. Measurements were conducted by investigators blinded to group allocation according to predefined measurement rules. Individual biological replicates are presented as individual data points in the graphs.

### 2.5. Western Blotting

Western blot analysis was performed to evaluate the expression levels of target proteins involved in cell death pathways. Total protein was extracted from myocardial tissue samples using RIPA buffer supplemented with protease and phosphatase inhibitors. Protein concentration was determined using the BCA assay. Equal amounts of protein (30 µg) were separated by 10% SDS-PAGE and subsequently transferred onto polyvinylidene fluoride (PVDF) membranes.

Membranes were blocked with 5% non-fat dry milk in Tris-buffered saline with 0.1% Tween-20 (TBST, Mill Creek, WA, USA) for 1 h at room temperature to prevent non-specific binding. The membranes were then incubated overnight at 4 °C with primary antibodies specific to the proteins of interest, including MLKL (ab187091, Abcam), p-MLLK (ab196436, Abcam), RIP3 (15828S, CST), Caspase-8 (9429S, CST), GSDMD (ab209845, Abcam), and GAPDH as a loading control. After washing with TBST, the membranes were incubated with appropriate horseradish peroxidase-conjugated secondary antibodies for one 1 h at room temperature.

Protein bands were visualized using an enhanced chemiluminescence (ECL) detection system. Densitometric analysis of Western blot bands was performed using ImageJ software, and band intensities were normalized to GAPDH and expressed relative to the control group.

Apoptosis was evaluated by detecting cleaved caspase-3 and related apoptotic markers, whereas pyroptosis signaling was assessed using established markers including cleaved caspase-1 and GSDMD-related signaling components.

### 2.6. Immunoprecipitation and Ubiquitination Assay

Neonatal mouse cardiomyocytes were transfected with MLKL siRNA or negative control siRNA for 48 h. To inhibit proteasomal degradation and allow accumulation of ubiquitinated proteins, cells were treated with the proteasome inhibitor MG132 (5 μM; Sigma-Aldrich, St. Louis, MO, USA) for 6 h prior to harvest. Cells were lysed in IP lysis buffer supplemented with protease inhibitors, phosphatase inhibitors, and N-ethylmaleimide (NEM, 10 mM; Sigma-Aldrich) to inhibit deubiquitinase activity.

Equal amounts of protein lysates were incubated overnight at 4 °C with anti-RIP3 antibody, followed by incubation with protein G agarose beads to immunoprecipitate RIP3 complexes. The immunoprecipitates were washed extensively and subjected to SDS-PAGE and immunoblotting using anti-ubiquitin and anti-RIP3 antibodies to evaluate RIP3 ubiquitination levels. Input lysates were analyzed in parallel to verify MLKL knockdown efficiency and total RIP3 protein expression.

### 2.7. Echocardiography

A Vevo 3100 high-resolution ultrasound system (FUJIFILM VisualSonics, Toronto, ON, Canada) equipped with a 30 MHz transducer utilized for imaging. Mice were positioned in the supine or left lateral decubitus position to facilitate access to the cardiac apex. Standard echocardiographic views, including parasternal long-axis and short-axis, were obtained to assess left ventricular (LV) dimensions and function.

Measurements included left ventricular ejection fraction (EF) and fractional shortening (FS), which were calculated from M-mode tracings using the Teichholz formula. Multiple cardiac cycles were recorded for each animal to obtain representative measurement 28 days after MI. Echocardiographic analyses were performed by investigators blinded to genotype and treatment allocation.

Images were acquired and analyzed using the Vevo LAB software 2100 (FUJIFILM VisualSonics), ensuring consistency in image acquisition settings across all samples. Multiple cardiac cycles were recorded to obtain representative measurements, and operator blinding was maintained to reduce bias. Post-echocardiography, mice were monitored until recovery from anesthesia and then returned to their respective housing conditions.

### 2.8. Study Approval

Male mice of 8 to 12 weeks were used in the experiments. The animal study protocol was approved by the Animal Care and Use Committee of Xiamen University (protocol code MMGLAC 20260101001, date: 1 September 2025).

### 2.9. Statistical Analysis

Statistical analyses were performed using GraphPad Prism (version 8.02) and SPSS (version 27.0). Data are presented as mean ± SEM unless otherwise specified. Comparisons between two groups were performed using an unpaired two-tailed Student’s *t*-test. Survival analyses were conducted using the Kaplan–Meier method with differences assessed by the log-rank (Mantel–Cox) test. A *p* value < 0.05 was considered statistically significant. The exact sample size (n) for each experiment is indicated in the corresponding figure legends. For in vitro experiments, n represents independent biological replicates; for in vivo experiments, n represents individual animals.

## 3. Result

### 3.1. Up Regulation of MLKL Expression in Hypoxia and MI Models

**Figure 1:**
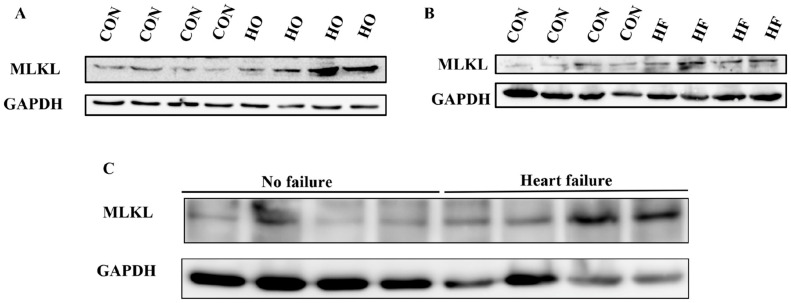
Upregulation of MLKL (Mixed Lineage Kinase Domain-Like protein) expression in hypoxia and myocardial infarction models. (**A**) Representative Western blot analysis of MLKL expression in H9C2 cardiomyocytes under control (CON) and hypoxic (HO) conditions. Quantification was performed from three independent biological replicates (*n* = 3). (**B**) Representative Western blot analysis of MLKL expression in myocardial tissue from mice 28 days after LAD ligation-induced MI. (**C**) Representative Western blot analysis of MLKL expression in human myocardial tissue samples. Human cardiac tissues were obtained from atrial appendage specimens of patients with rheumatic valvular heart disease, including non-failing (No failure) and heart failure (Heart failure) groups. Quantification was performed from *n* = 3 independent biological replicates. Band intensities were normalized to GAPDH where applicable. Data are presented as mean ± SEM. CON, normoxic control; HO, hypoxia; No failure, non-failing human atrial appendage tissue; Heart failure, atrial appendage tissue from patients with heart failure secondary to rheumatic heart disease.

To gain deeper insights into the pathological role of MLKL in HF, we established both an H9C2 cell hypoxia model and an MI mouse model. Our findings revealed an upregulation of MLKL expression in H9C2 cardiomyocytes under hypoxic (HO) conditions compared to normoxic control (CON) (Figure 1A). MLKL expression was also elevated in the myocardial tissue of mice 28 days after MI (Figure 1B). MLKL levels were increased in human myocardial tissue samples derived from atrial appendage specimens of patients with rheumatic valvular heart disease, including non-failing and heart failure groups (Figure 1C). These findings collectively suggest that MLKL may be involved in pathological processes associated with myocardial injury and heart failure.

### 3.2. MLKL Deficiency Exacerbates Myocardial Injury, Functional Decline, and Cardiac Fibrosis Following MI

**Figure 2:**
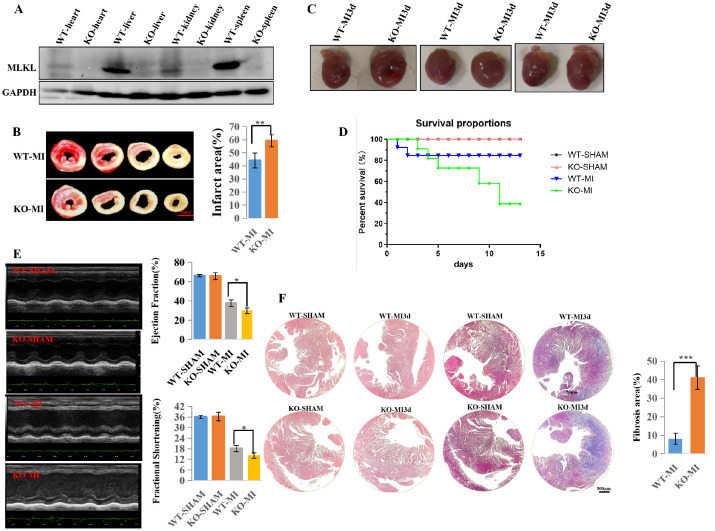
MLKL deficiency is associated with increased myocardial injury, functional decline, and cardiac fibrosis after myocardial infarction (MI). (**A**) Validation of MLKL deletion in wild-type (WT) and MLKL knockout (KO) mouse hearts by Western blot analysis. (**B**) Representative TTC (triphenyltetrazolium chloride) staining and quantification of infarct size in WT-MI and KO-MI hearts 3 days after MI (*n* = 3 mice per group). (**C**) Representative gross morphology of hearts 3 days after MI. (**D**) Kaplan–Meier survival analysis comparing WT and MLKL-KO mice after MI. WT-SHAM (*n* = 7), KO-SHAM (*n* = 7), WT-MI (*n* = 15), and KO-MI (*n* = 15). (**E**) Echocardiographic assessment of cardiac function, including left ventricular ejection fraction (LVEF) and fractional shortening (FS), was performed at 28 days after myocardial infarction (MI). Echocardiographic measurements were analyzed in a blinded manner. (**F**) Representative hematoxylin and eosin (H&E) and Masson’s trichrome staining showing myocardial injury and fibrosis 3 days after MI, with corresponding quantitative analysis. Quantification was performed from *n* = 3 independent biological replicates. Data are presented as mean ± SEM. Statistical significance was determined using an unpaired two-tailed Student’s *t*-test. ns, *p* ≥ 0.05; * *p* < 0.05; ** *p* < 0.01; *** *p* < 0.001. SHAM, sham-operated; MI3d, 3 days after MI.

To further investigate the molecular mechanisms underlying MLKL’s role in HF, we generated an MLKL knockout (MLKL^−/−^, KO) MI mouse model. Successful deletion of the MLKL gene was confirmed by Western blot analysis (Figure 2A). TTC staining revealed a marked increase in infarct size in KO-MI compared to WT-MI at 3 days after MI (Figure 2B; *n* = 3 per group), indicating that MLKL deficiency may exacerbate myocardial injury. Representative gross morphology demonstrated increased cardiac enlargement in KO-MI compared to WT-MI at 3 days after MI (Figure 2C). Kaplan–Meier survival analysis demonstrated reduced survival in KO-MI compared to WT-MI (Figure 2D; WT-SHAM *n* = 7, KO-SHAM *n* = 7, WT-MI *n* = 15, KO-MI *n* = 15), further supporting the detrimental role of MLKL in MI and HF pathogenesis.

To evaluate cardiac function, echocardiographic analysis was performed at 28 days after MI (Figure 2E). Both WT-MI and KO-MI groups exhibited reduced left ventricular ejection fraction (LVEF) and fractional shortening (FS), with a more pronounced decline in the KO-MI group (Figure 2E). However, the KO-MI group demonstrated a more pronounced decline in these parameters compared to the WT-MI group, suggesting that blocking MLKL-mediated programmed necrosis worsens myocardial dysfunction and exacerbates post-MI cardiac failure.

Histological analysis performed at 3 days after MI demonstrated more severe myocardial structural disruption and fibrosis in KO-MI compared to WT-MI (Figure 2F). Both the KO-MI and WT-MI groups exhibited loosening of interstitial spaces and disruption of myocardial fibers; However, the KO-MI exhibited more severe tissue damage, with greater interstitial loosening and more pronounced myocardial fiber disruption (Figure 2F). Additionally, myocardial fibrosis was more extensive in the KO-MI compared to the WT-MI, indicating that MLKL deficiency leads to greater myocardial scarring. Taken together, these results demonstrate that the knockout of the MLKL gene, by inhibiting the RIP3-MLKL-mediated programmed necrosis pathway, does not alleviate myocardial injury following MI but rather exacerbates myocardial cell death, further impairing heart function and promoting cardiac fibrosis, indicating that the essential role of MLKL in mitigating myocardial damage and preserving cardiac function after MI, in contrast to the phenotype observed following RIP3 ablation [1] or RIP1 inhibition [3].

### 3.3. MLKL Deficiency Increases RIP3 Protein Levels After MI Independent of Transcriptional Regulation

Different regulated cell death modalities share overlapping signaling components and, under specific stress conditions, can interconvert [4,5,6]. Consequently, inhibiting a single death pathway does not necessarily alter the fate of the cell, as it may switch to an alternative death program when one route is blocked. To further investigate the role of MLKL in regulating RIP3 expression, we assessed RIP3 protein levels in hypoxia-stimulated neonatal mouse cardiomyocytes (NMCs) and in myocardial tissue from MI mice.

**Figure 3:**
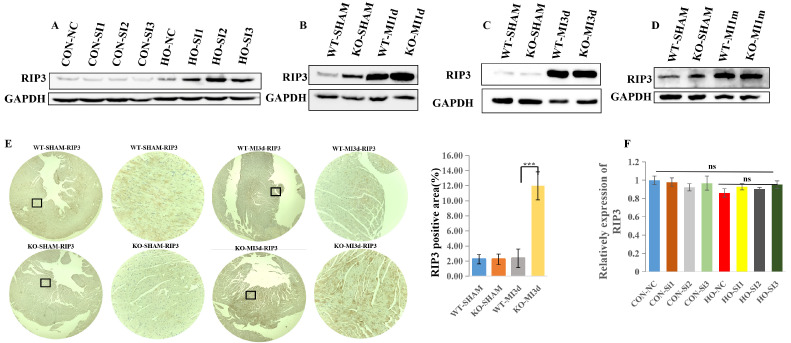
MLKL Deficiency Enhances RIP3 Protein Accumulation Following Myocardial Infarction (**A**) Representative Western blot and corresponding quantification of RIP3 protein expression in negative control (NC) and MLKL-silenced cardiomyocytes under hypoxic conditions. (**B**–**D**) Representative Western blots and quantification of RIP3 protein expression in WT-MI and KO-MI hearts at the indicated post-MI time points. The enlarged image of the small black box is shown on its right. (**E**) Representative histological or immunostaining assessment of RIP3 expression in myocardial tissue, with quantification where applicable. Quantitative real-time PCR analysis of RIP3 mRNA expression (**F**). Quantification was performed from *n* = 3 independent biological replicates. Band intensities were normalized to GAPDH where applicable. Data are presented as mean ± SEM. Statistical significance was determined using the unpaired two-tailed Student’s *t*-test. ns, *p* ≥ 0.05; *** *p* < 0.001. SI, small interfering RNA; MI1m, 1 month after MI.

As shown in Figure 3A, RIP3 protein expression was elevated under hypoxic conditions in both the negative control (NC) and MLKL siRNA (SI) groups. Notably, RIP3 protein expression was higher in the MLKL siRNA (SI) group compared with the NC group under hypoxic conditions (Figure 3A). In the MI mouse model, RIP3 protein levels were also upregulated in both WT and KO groups. However, RIP3 protein expression was higher in KO-MI hearts compared with WT-MI hearts at the indicated post-MI time points (Figure 3B,C).

Immunohistochemical analysis further confirmed increased RIP3 protein expression in KO-MI hearts compared with WT-MI hearts (Figure 3E). Building on our observation that MLKL knockdown augments RIP3 expression after MI, we first assessed whether this increase reflects transcriptional regulation.

Quantitative real-time PCR showed that MLKL silencing did not increase RIP3 mRNA expression under basal or hypoxic conditions, suggesting that the observed increase in RIP3 protein abundance after MI and hypoxia is likely regulated at the post-transcriptional level (Figure 3F).

### 3.4. MLKL Knockdown Stabilizes RIP3 by Attenuating Its Ubiquitin–Proteasome-Mediated Degradation in NMCs

**Figure 4:**
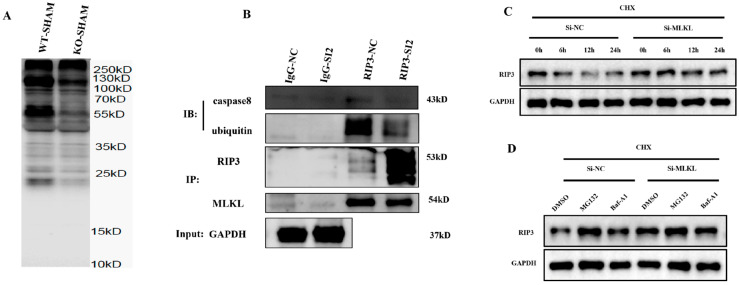
MLKL Deficiency Increases RIP3 Protein Stability in Neonatal Mouse Cardiomyocytes (NMCs) (**A**–**D**) Representative immunoblot analyses illustrating RIP3 expression, ubiquitination, and protein stability in control and MLKL-silenced cardiomyocytes under the indicated experimental conditions. Cycloheximide-chase experiments were used to evaluate RIP3 protein turnover. Where indicated, cells were treated with the proteasome inhibitor MG132 to assess ubiquitin–proteasome-dependent degradation. Data represent three independent experiments (*n* = 3). Band intensities were normalized to GAPDH where applicable. Data are presented as mean ± SEM. Statistical significance was determined using an unpaired two-tailed Student’s *t*-test. DSMO, Dimethyl Sulfoxide; CHX, Cycloheximide.

MLKL knockdown reduced ubiquitin conjugation of RIP3 under proteasome-inhibited conditions, while total RIP3 protein levels were increased in the input fraction (Figure 4A,B).

In cycloheximide (CHX) chase assays, MLKL knockdown prolonged RIP3 protein persistence. This stabilization was phenocopied and occluded by the proteasome inhibitor MG132 (5 µM) but was insensitive to lysosomal blockade with bafilomycin A1 (Baf-A1, 50 nM), indicating selective impairment of proteasome- mediated turnover (Figure 4C,D). These data indicate that loss of MLKL is associated with increased RIP3 abundance through proteasomal-mediated degradation.

### 3.5. MLKL Knockdown Enhances Apoptotic Signaling in Hypoxia-Treated Cardiomyocytes

**Figure 5:**
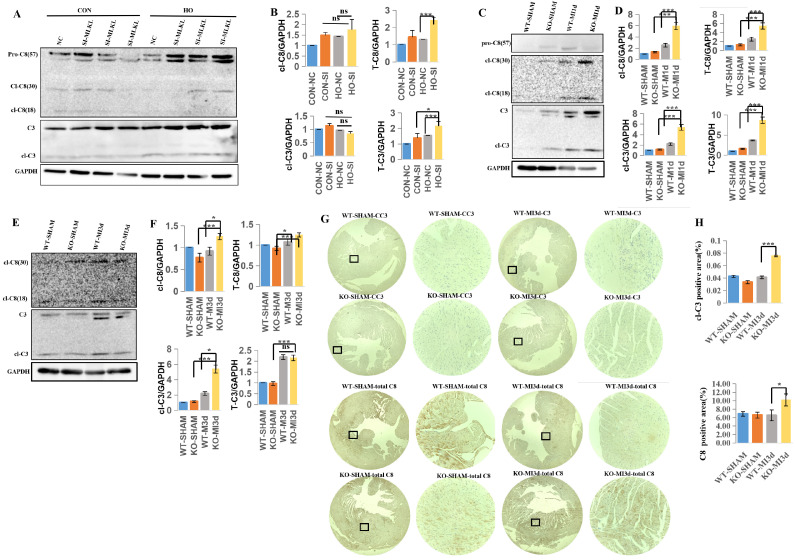
MLKL deficiency is associated with enhanced activation of apoptosis-related signaling following myocardial infarction. (**A**,**B**) Representative Western blot analyses and corresponding quantification of apoptosis-related proteins in control and MLKL-silenced cardiomyocytes under hypoxic conditions. (**C**,**D**) Representative immunoblot analyses of apoptosis-related proteins in myocardial tissue from WT-MI and MLKL-KO MI mice at 1 day after myocardial infarction. (**E**,**F**) Representative immunoblot analyses of apoptosis-related proteins in myocardial tissue from WT-MI and MLKL-KO MI mice at 3 days after myocardial infarction. (**G**,**H**) Representative immunostaining images and quantitative analysis of apoptosis markers in myocardial tissue sections. The enlarged image of the small black box is shown on its right. Data represent three independent biological replicates for cell experiments. WT-SHAM (*n* = 7), KO- SHAM (*n* = 7), WT-MI (*n* = 13), and KO-MI (*n* = 13) groups. Band intensities were normalized to GAPDH. Data are presented as mean ± SEM. Statistical significance was determined using an unpaired two-tailed Student’s *t*-test. ns, *p* ≥ 0.05; * *p* < 0.05; *** *p* < 0.001. CC3, Cleaved Caspase-3; C8, Cysteine-aspartic protease 8.

Following hypoxic stimulation, both the negative control (NC) and MLKL siRNA (SI) groups exhibited increased expression of apoptosis-related proteins. Notably, the SI group showed higher levels of cleaved Caspase-8 (cl-Caspase-8) and Caspase-3 compared with the NC group (Figure 5A,B), indicating enhanced activation of apoptosis-related signaling under MLKL knockdown conditions.

In the MI mouse model, apoptosis-related protein expression was increased in both WT-MI and KO-MI groups at 1 day after MI. Higher levels of Caspase-8, cleaved Caspase-8, Caspase-3, and cleaved Caspase-3 were observed in KO-MI hearts compared with WT-MI hearts at this time point (Figure 5C,D). At 3 days after MI, both groups exhibited elevated expression of apoptosis-related proteins, with no apparent difference between KO-MI and WT-MI hearts (Figure 5E,F).

Immunohistochemical analysis further demonstrated increased expression of apoptosis-related markers, including Caspase-8 and cleaved Caspase-3, in KO-MI hearts compared with WT-MI hearts (Figure 5G,H).

Taken together, these findings indicate that MLKL deficiency is associated with enhanced activation of apoptosis-related signaling following MI, particularly at early time points.

### 3.6. MLKL Knockdown Enhances Pyroptotic Signaling in Hypoxia-Treated Cardiomyocytes

**Figure 6:**
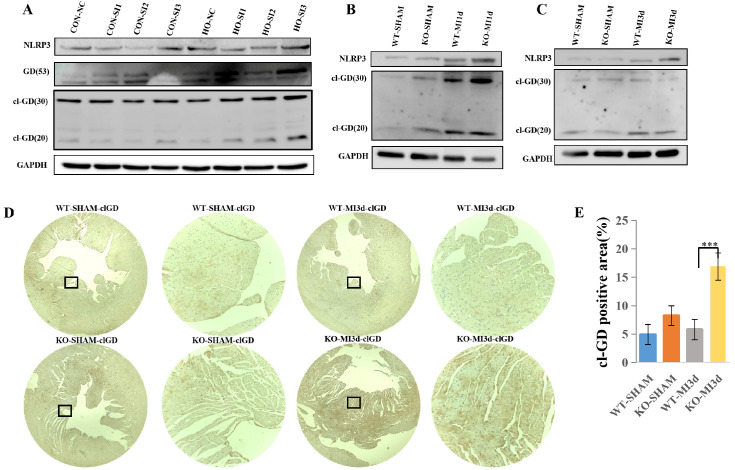
MLKL Deficiency Is Associated with Enhanced Pyroptosis Signaling Following Myocardial Infarction (**A**) Representative Western blot analysis and corresponding quantification of pyroptosis-related proteins in control and MLKL-silenced cardiomyocytes under hypoxic conditions. (**B**) Representative immunoblot analyses of pyroptosis pathway proteins in myocardial tissue from WT-MI and MLKL-KO MI mice at 1 day after myocardial infarction. (**C**) Representative immunoblot analyses of pyroptosis pathway proteins in myocardial tissue from WT-MI and MLKL-KO MI mice at 3 days after myocardial infarction. (**D**,**E**) Representative immunostaining images and quantitative analysis of pyroptosis markers in myocardial tissue sections. The enlarged image of the small black box is shown on its right. *n* values: MI1d, WT-SHAM (*n* = 7), KO-SHAM (*n* = 7), WT-MI (*n* = 15), KO-MI (*n* = 15); MI3d, WT-SHAM (*n* = 7), KO-SHAM (*n* = 7), WT-MI (*n* = 13), KO-MI (*n* = 13). Quantification was performed from *n* = 3 independent biological replicates where applicable. Band intensities were normalized to GAPDH. Data are presented as mean ± SEM. Statistical significance was determined using an unpaired two-tailed Student’s *t*-test. *** *p* < 0.001. SI1/SI2/SI3, Small interfering RNA sequences 1, 2, and 3; NLRP3, NLR Family Pyrin Domain Containing 3; GD, Gasdermin D; cl-GD, Cleaved Gasdermin D.

Following hypoxic stimulation, cleaved Gasdermin D (cl-GD) expression was increased in MLKL siRNA (SI) cells compared with negative control (NC) cells (Figure 6A), indicating enhanced pyroptosis-related signaling under MLKL knockdown conditions.

In the MI model, pyroptosis-related protein expression was increased in both WT-MI and KO-MI groups at 1 day after MI. Higher levels of NLRP3 and cleaved Gasdermin D (cl-GD) were observed in KO-MI hearts compared with WT-MI hearts at this time point (Figure 6B). At 3 days after MI, both groups exhibited increased expression of NLRP3 and GSDMD, with no apparent difference between KO-MI and WT-MI hearts (Figure 6C).

Immunohistochemical analysis further demonstrated increased cl-GD expression in KO-MI hearts compared with WT-MI hearts (Figure 6D,E).

Taken together, these findings indicate that MLKL deficiency is associated with enhanced activation of pyroptosis-related signaling following MI, particularly at early time points.

### 3.7. RIP3 Inhibition by GSK’872 Reverses Excessive Apoptosis and Pyroptosis Activation and Alleviates Cardiac Dysfunction in MLKL KO Mice Following MI

**Figure 7:**
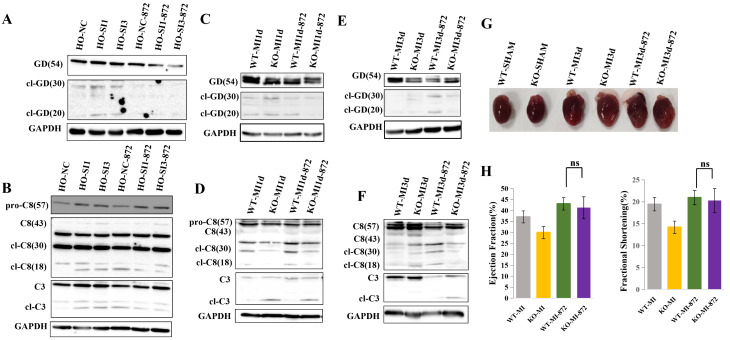
RIP3 inhibition by GSK’872 is associated with attenuation of excessive apoptosis and pyroptosis signaling and improvement of cardiac function in MLKL-KO mice following myocardial infarction (MI). (**A**,**B**) Representative Western blot analyses and corresponding quantification of apoptosis-related and pyroptosis-related proteins in myocardial tissues from WT-MI and MLKL-KO MI mice treated with vehicle or the RIP3 inhibitor GSK’872. (**C**,**D**) Representative immunoblot analyses showing modulation of downstream signaling molecules involved in apoptosis and pyroptosis pathways following RIP3 inhibition 1 day after myocardial infarction (MI1d). (**E**,**F**) Representative immunoblot analyses showing modulation of apoptosis and pyroptosis markers in myocardial tissue following GSK’872 treatment 3 days after myocardial infarction (MI3d). (**G**,**H**) Echocardiographic assessment of cardiac function including left ventricular ejection fraction (LVEF) and fractional shortening (FS) in WT-MI and MLKL-KO MI mice with or without GSK’872 treatment. A single intraperitoneal dose of GSK’872 (1.9 mg/kg) was administered before LAD ligation. Data represent three independent biological replicates for cell-based experiments unless otherwise indicated. *n* values: MI1d, WT-SHAM (*n* = 7), KO-SHAM (*n* = 7), WT-MI (*n* = 15), KO-MI (*n* = 15); MI3d, WT-SHAM (*n* = 7), KO-SHAM (*n* = 7), WT-MI (*n* = 13), KO-MI (*n* = 13). Band intensities were normalized to GAPDH where appropriate. Data are presented as mean ± SEM. Statistical significance was determined using an unpaired two-tailed Student’s *t*-test. ns, *p* ≥ 0.05.

Under hypoxic conditions, GSK’872 [7] treatment (1.9 mmol/kg, via intraperitoneal injection to both the WT-MI and KO-MI groups [8]) was associated with reduced expression of apoptosis- and pyroptosis-related proteins in both HO-NC and HO-SI groups. Lower levels of cleaved Gasdermin D (cl-GD), cleaved Caspase-8, and cleaved Caspase-3 were observed in the HO-SI-872 group compared with the HO-NC-872 group (Figure 7A,B).

In the MI model, GSK’872 treatment was associated with reduced expression of apoptosis- and pyroptosis-related proteins in both WT-MI and KO-MI groups. Lower levels of cleaved Gasdermin D (cl-GD) were observed in KO-MI-872 hearts compared with WT-MI-872 hearts (Figure 7C,E). In addition, cleaved Caspase-8 expression was reduced in KO-MI-872 hearts compared with WT-MI-872 hearts (Figure 7D,F).

Echocardiographic and representative gross morphology assessment showed improved cardiac function in both WT-MI and KO-MI groups following GSK’872 treatment. No apparent differences in cardiac function parameters were observed between KO-MI-872 and WT-MI-872 groups (Figure 7G,H).

Taken together, these findings indicate that RIP3 inhibition by GSK’872 is associated with reduced apoptosis- and pyroptosis-related signaling and improved cardiac function after MI, including under MLKL-deficient conditions.

### 3.8. Mechanistic Illustration of MLKL-Mediated Regulation of RIP3 and Caspase-8 Interaction in the Activation of Apoptosis and Pyroptosis Pathways Following MI

**Figure 8:**
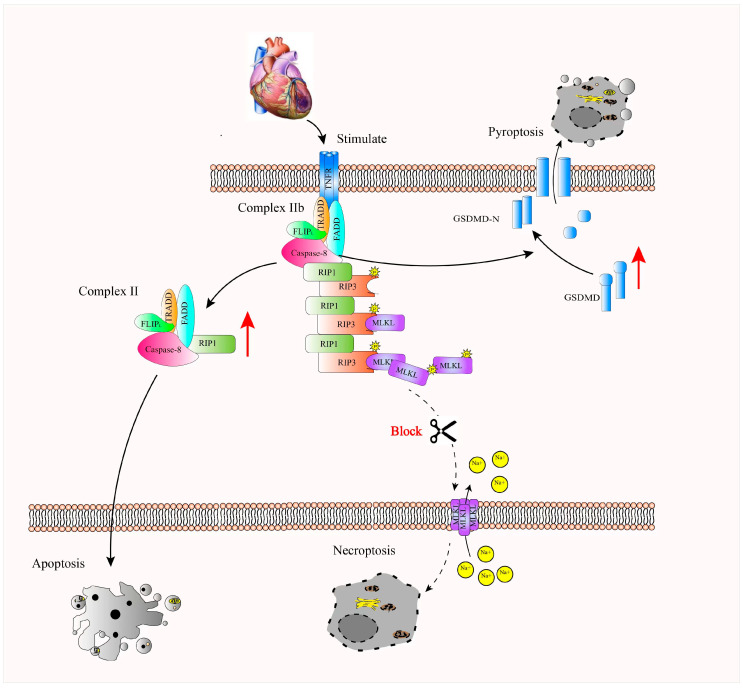
Proposed mechanistic model summarizing the role of MLKL–RIP3 signaling in regulating apoptosis and pyroptosis following myocardial infarction (MI). In this working model, MLKL deficiency is associated with increased RIP3 protein stability, potentially through reduced ubiquitin–proteasome-mediated degradation. Elevated RIP3 levels are proposed to enhance activation of apoptosis-related and pyroptosis-related signaling pathways in cardiomyocytes. The resulting increase in cardiomyocyte death may contribute to aggravated myocardial injury and adverse cardiac remodeling following MI. This schematic integrates the experimental findings presented in Figure 1, Figure 2, Figure 3, Figure 4, Figure 5, Figure 6 and Figure 7 and provides a conceptual framework for the proposed MLKL–RIP3 regulatory axis in myocardial injury. A red upward arrow indicates an increase in the activation of the corresponding pathway.

MLKL deficiency is associated with increased RIP3 protein levels, which may involve interaction with Caspase-8, potentially contributing to activation of cleaved Caspase-3 and Gasdermin D. This is associated with enhanced apoptosis- and pyroptosis-related signaling, which may contribute to increased cardiomyocyte death following MI. Our findings suggest that apoptosis and pyroptosis are interconnected processes in the context of MI. Furthermore, these results suggest a potential role for MLKL in this regulatory network and suggesting that MLKL–RIP3 signaling may represent a potential therapeutic target in MI (Figure 8).

## 4. Discussion

MI remains a leading cause of sudden cardiac death, with myocardial cell death being a central event in its pathogenesis. Historically, extensive research has focused on the role of apoptosis in MI pathogenesis, with early studies establishing its critical role in myocardial injury [9,10,11,12,13]. However, more recent publications have called into question the primacy of apoptosis in MI [14], with increasing evidence suggesting that other forms of cell death, such as [15], programmed necrosis [16,17,18], ferroptosis [19,20,21], pyroptosis [22], PARP-dependent cell death [23], and autophagy-dependent cell death [24,25], also contribute significantly to myocardial cell loss. Current research highlights that the majority of acute cell death in MI is mediated by necroptosis pathways [26,27], making a thorough understanding of these mechanisms crucial for identifying effective therapeutic targets. Making a more comprehensive understanding of these mechanisms important for identifying potential therapeutic targets.

MLKL, a substrate of RIP3 kinase, is a key executioner protein in the programmed necrosis pathway [28]. Programmed necrosis, including RIP3-mediated necroptosis, has been implicated as a significant component of the pathophysiology of various cardiovascular diseases, including atherosclerosis, myocardial IR injury, MI, and cardiac remodeling [29,30]. Thus, further investigation of the function and regulatory mechanisms of MLKL may provide additional insights into MI pathogenesis.

In this study, we generated an MLKL^−/−^ mouse model of MI to investigate the function of MLKL in the context of myocardial injury. Compared to WT-MI mice, KO-MI exhibited a trend towards increased infarct size, more pronounced cardiac hypertrophy post-infarction, significantly worsened cardiac function, greater myocardial fiber disarray, and more extensive fibrosis, in contrast to the phenotype observed following RIP3 ablation or RIP1 inhibition.

In the present study, we provide evidence that MLKL regulates RIP3 abundance at the post-translational level rather than through transcriptional control. Although MLKL is classically viewed as a terminal executioner of necroptosis downstream of RIP3 [31,32], our data reveal an unexpected upstream regulatory role of MLKL in modulating RIP3 protein stability. Specifically, MLKL deficiency resulted in reduced ubiquitination of RIP3 and impaired proteasome-mediated degradation, leading to sustained accumulation of RIP3 following hypoxic stress and MI. This finding expands the current understanding of necroptotic signaling by suggesting that MLKL participates in a feedback regulatory loop that constrains RIP3 abundance under physiological conditions. Our data suggest that MLKL may influence RIP3 protein abundance at the post-translational level rather than through transcriptional regulation. This observation further supports a potential regulatory interaction within necroptotic signaling pathways.

It is well-documented that various cell death pathways are interconnected, with each pathway potentially influencing or complementing others in a dynamic cascade [33,34,35,36]. For example, Zhang et al. [37] described how the NLRC4 inflammasome induces cell death via three complementary death pathways: pyroptosis mediated by Caspase-1-GSDMD, apoptosis through ASC-mediated Caspase-8 activation [37,38], and an intrinsic apoptotic pathway triggered by Caspase-1 in the absence of pyroptosis and apoptosis. Mice deficient in one or two of these pathways still succumb to death [39], but complete inhibition of all death pathways prevents NLRC4-mediated cell death and tissue damage. This suggests that different cell death pathways can compensate for each other in specific contexts, making their precise regulation crucial for maintaining tissue homeostasis.

Previous research [5] has shown that RIP3 serves as a molecular switch that regulates the transition between TNF-induced apoptosis and programmed necrosis, with cellular energy metabolism influencing the choice of cell death modality. These findings suggest that RIP3 may play a pivotal role in determining whether a cell undergoes apoptosis, pyroptosis, or necroptosis following MI.

In line with this, we hypothesize that MLKL knockout exacerbates cardiac dysfunction and increases mortality following MI by triggering the activation of alternative cell death pathways. Indeed, our findings show that MLKL silencing leads to excessive activation of both apoptosis and pyroptosis pathways at different stages of MI, which could explain the reduced survival rates and more severe cardiac dysfunction observed in the KO-MI mice. These results point to a critical role for MLKL in regulating the balance between cell death pathways and suggest that its inhibition may disrupt this balance, thereby promoting excessive myocardial injury. These results suggest that MLKL may participate in regulating the balance between multiple cell death pathways.

In this study, treatment with GSK’872, a potent and selective RIP3 kinase inhibitor, significantly mitigated cardiac dilation and functional deterioration in MLKL-deficient mouse models following MI. Moreover, GSK’872 treatment reversed the marked upregulation of proteins associated with both apoptosis and pyroptosis pathways. These findings suggest that MLKL knockout or silencing leads to an increase in RIP3 levels, which, in turn, promotes apoptosis and pyroptosis through the RIP3-Caspase-8 axis. Inhibition of RIP3 kinase activity by GSK’872 effectively suppresses the excessive activation of both apoptotic and pyroptotic pathways, which is triggered by MLKL inhibition.

Overall, these results reinforce the idea that MLKL-mediated regulation of RIP3 plays a crucial role in modulating cell death pathways in the context of MI. The ability of GSK’872 to reverse the detrimental effects of MLKL knockout further highlights the potential therapeutic utility of targeting RIP3 in preventing the excessive activation of apoptosis and pyroptosis, thereby alleviating myocardial injury and improving heart function. Suggests that targeting RIP3 signaling pathways may represent a potential therapeutic strategy.

Our study acknowledges several limitations that should be considered. First, the precise molecular mechanisms governing the interaction between RIP3 and other death pathways, including the identification of specific binding sites, remain an area of ongoing investigation. The network of programmed cell death pathways is highly complex, and this study provides only a preliminary exploration of its components, focusing on a limited subset of interactions.

In addition, the precise regulatory role of RIP3 across these three distinct cell death pathways likewise requires further mechanistic elucidation, particularly within cardiovascular disease models.

Further investigations are required to fully elucidate the precise mechanism by which RIP3 expression is upregulated following MLKL knockout and warrants additional investigation to delineate the underlying regulatory network. These insights may ultimately reveal new therapeutic targets to modulate cell death pathways and mitigate myocardial injury in conditions such as MI.

## 5. Conclusions

Our data further reveal that MLKL knockout or silencing does not mitigate cardiac damage post-MI. Rather than improving outcomes, MLKL deficiency exacerbates myocardial injury by significantly upregulating both pyroptosis and apoptosis pathways, which contributes to worsened cardiac function and increased mortality. Our findings suggest that MLKL deficiency does not alleviate myocardial injury following MI.

The interconnectivity of programmed cell death pathways-programmed necrosis, apoptosis, and pyroptosis was highlighted within myocardial cells. These pathways appear to be intricately linked, and their combined activation following MLKL inhibition leads to more severe outcomes in MI.

Importantly, our findings also demonstrate that the RIP3 inhibitor GSK’872 can effectively counteract the excessive activation of apoptosis and pyroptosis pathways induced by MLKL deficiency. This suggests that targeting RIP3 may be a viable strategy to attenuate myocardial injury and improve cardiac outcomes following MI.

In conclusion, our study provides compelling evidence that MLKL plays a critical role in regulating cell death pathways in MI. Therapeutic strategies aimed at modulating MLKL expression or inhibiting RIP3 kinase activity could offer new avenues for the treatment of MI and the prevention of post-infarction heart failure. Our study provides evidence supporting a regulatory role of MLKL in multiple cell death pathways during MI.

## Data Availability

The data presented in this study are available from the corresponding author upon reasonable request.
